# Investigation on parasitic infestation of *Labeo rohita* (Hamilton, 1822) in the south‐western region of Bangladesh

**DOI:** 10.1002/vms3.1489

**Published:** 2024-06-12

**Authors:** Basir Ahammad, Bhaskar Chandra Majumdar, Angkur Chowdhury, Rasel Mia, MD Zobayer Rahman

**Affiliations:** ^1^ Department of Oceanography Khulna Agricultural University Khulna Bangladesh; ^2^ Department of Fishery Biology and Genetics Khulna Agricultural University Khulna Bangladesh; ^3^ Department of Aquatic Resource Management Sylhet Agricultural University Sylhet Bangladesh; ^4^ Department of Fish Health Management Sylhet Agricultural University Sylhet Bangladesh

**Keywords:** Parasite, Histology, *Labeo rohita*, Bangladesh

## Abstract

**Background:**

Our investigation focused into *Labeo rohita*, commonly known as Rui, a freshwater aquatic species in Bangladesh. Despite their nutritional significance, these fish faced a pressing challenge: parasite infections threaten the economic stability of the aquaculture sector.

**Objectives:**

The present study aimed to investigate the parasite and histological changes in major organs of *L. rohita*, collected from Khulna region – Dumuria, Paikgacha and Rupsha.

**Methods:**

About 180 (30/month) specimens were collected between the month of March and August 2023 to observe the parasitic status in *L. rohita*.

**Results:**

Through microscopic examination, a total of 323 parasites were uncovered, spanning categories including Cestode, Nematode, Acanthocephala, Trematode and Digenia, predominantly residing in the intestines of *L. rohita*. The highest prevalence rate (70%) was recorded in both March and May 2023, with peak mean intensity observed in July (3.73). Notably, the highest mean abundance (2.37) exhibited in July and index of infestation (45.34) in June. Histological analysis confirmed parasitic infestations in the gastrointestinal region, with displaying histological changes within major organs such as the liver, kidney, gills, spleen and testicles due to parasitic infection.

**Conclusion:**

This study concluded that the indentified six categories of parasite and the affect of parasitic infestation in major organs of *L. rohita* within the study period. Urgent efforts to implement effective strategies for controlling the parasite infections in aquaculture to ensure the sustainable production of this invaluable fish species.

## Graphical abstract

Over the study, 180 *L. rohita* specimens were examined,, revealing the presence of six distinct parasite species, consisting of endoparasites. Through microscopic examination, a total of 323 parasites were investigated, categories including Cestode, Nematode, Acanthocephala, Trematode and Digenia, predominantly residing in the intestines and liver of *L. rohita*.

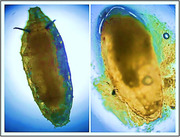

## 1 INTRODUCTION

Bangladesh stands as the world's largest riverine country, boasting a remarkable abundance of productive water resources and a wealth of diverse fisheries (Nasren et al., 2023; Zaman et al., 2023). Bangladesh holds immense potential in its fisheries sector, encompassing both its inland waters and the territorial seas, which have a rich array of fish species. The country's fishery resources are characterized by a diverse spectrum, including 260 native freshwater species, 12 exotic fish species, 24 freshwater prawn species, 475 marine fish species and 36 marine shrimp species (Rahman et al., 2023; Mia et al., 2024). In our nation, fisheries resources contribute significantly to our diet, providing approximately 63% of the animal protein consumed (Ahmed, 2005).

In our country, indigenous species like *Labeo rohita* have long been cherished for their delicious taste, comprise a significant portion of our culinary traditions since ancient times (Islam et al., 2014; Khan et al., 2006). The delectable flavour and palatability of fish to high demand in the market, greatly enhancing their commercial value (Khan et al., 2012). Parasitic infestation stands as a significant concern and formidable threat within both culture systems and natural aquatic habitats, endangering fish populations and presenting a substantial obstacle to the promotion and preservation of fish health (Upadhyay et al., 2012).

The adverse impacts of parasites on fish encompass a wide range of effects. Understanding parasitology holds significance in fishery management, optimizing fish yields and controlling the transmission of diseases to humans and animals, where fish often serve as carriers (Srivastava, 1975). The prevalence of *Clinostomum complanatum* in fish from the floodplain of the Upper Paraná River in Brazil was found to be unaffected by season, habitat or sex(Dias et al., 2006). There has been relatively limited research conducted on digenetic trematodes in Bangladesh, particularly in relation to the abundance of larval trematodes and the local benthic and fish populations (Hechinger et al., 2007).

*L. rohita* possesses a sleek, streamlined body adorned with sizable cycloid scales and a subtly flattened head. Its lateral line is notably prominent, running the entire length of its body. The fish features dorsal, pectoral, pelvic and anal fins, with the dorsal fin distinguished by a serrated spine (Ahsan et al., 2013). *L. rohita* frequently displays schooling behaviour, particularly in its early life stages. Juvenile have a propensity to assemble in sizable clusters, a strategy believed to provide collective protection (Azim et al., 2001). *L. rohita* renowned for its surface feeding habits and proves susceptible to a variety of feeding methods in aquaculture. Notably adaptable to a broad spectrum of temperatures, this species thrives in diverse aquatic habitats. With its modest oxygen requirements, it flourishes even in environments with moderate oxygen levels (Biswas et al., 2015). *L. rohita* is frequently cultivated in ponds, often in polyculture with other species such as catfish and carp, as part of integrated farming systems aimed at maximizing resource efficiency. In aquaculture, the fish is provided with a well‐balanced diet comprising both plant and animal proteins to promote optimal growth (Rahman & Arifuzzaman, 2021). The flesh of *L. rohita* holds great esteem in South Asian culinary traditions, greatly enhancing its market desirability. The aquaculture and fisheries sectors linked to *L. rohita* play a pivotal role in providing substantial employment opportunities for many individuals within the region (Islam & Hossain, 2013). Ongoing research endeavours focus on developing strategies to effectively manage diseases and parasitic infestations in both culture and wild populations (Jena et al., 2011).

The biology, behaviour and ecological aspects of *L. rohita* is crucial for implementing sustainable management approaches in aquaculture or the conservation of wild populations (Samada et al., 2017). Under optimal conditions of temperature, host physiology and water quality in rivers or ponds, parasites proliferate and multiply (Shomorendra et al., 2005).

However, in Bangladesh, studies about parasitic infestation in *L. rohita* in wild environment are limited. A few numbers of studies have been reported on the monitoring of parasitic infestation in *L. rohita* in south‐western region of Bangldesh. Thus, the present study was aimed to determine the presence of parasitic infestation in *L. rohita* collected from three areas of Khulna district in Bangladesh.

## 2 MATERIALS AND METHODS

### 2.1 Study area and time frame

**FIGURE 1:**
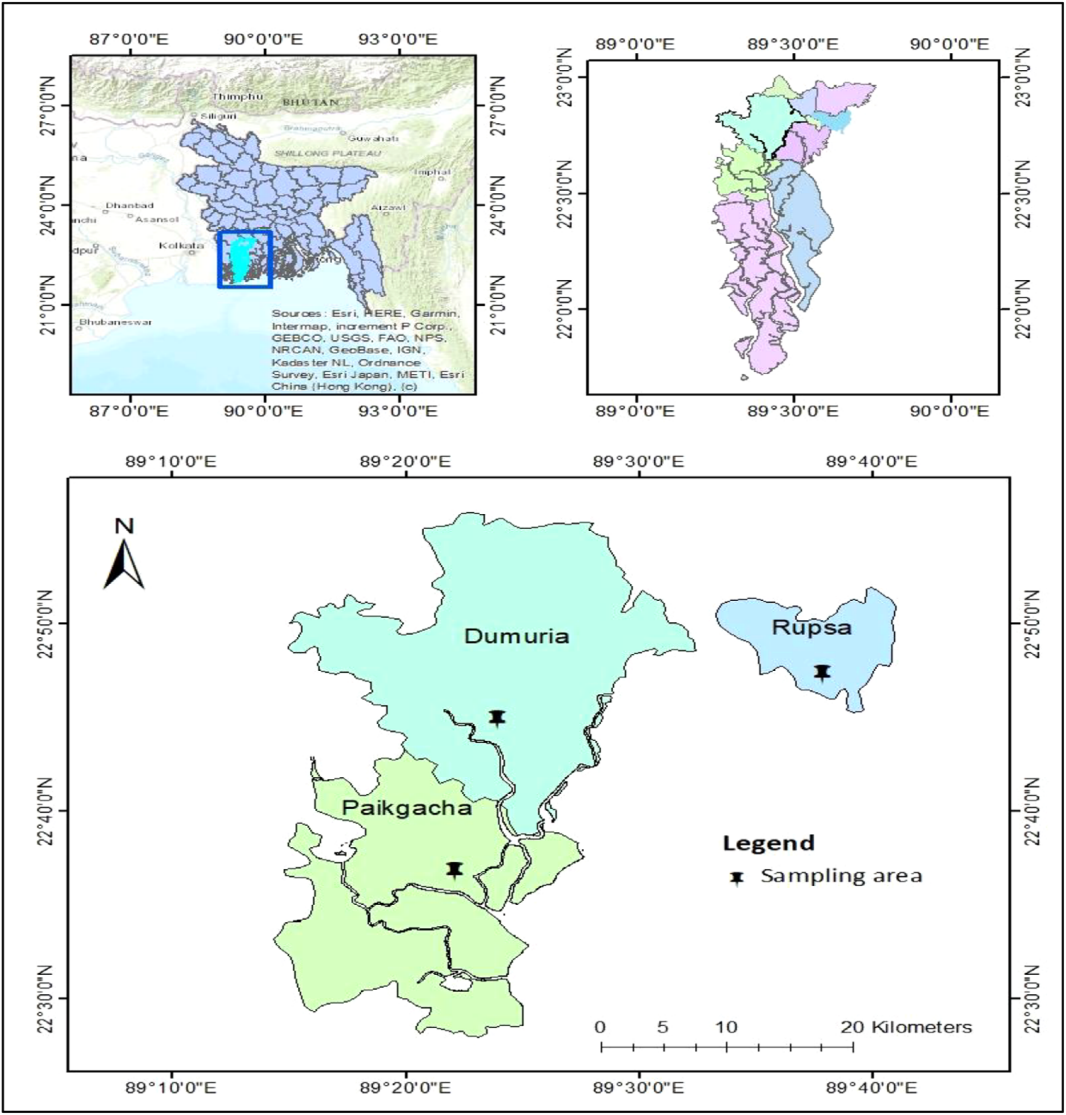
Map showing the study area. *Source*: Mapping software: ARC GIS, Version‐10.8.

About 180 *L. rohita* (30 fishes in per month) were collected from three distinct place of Khulna known as Dumuria, Paikgacha and Rupsha in the month of March 2023 to August 2023 (Figure 1). Then the samples were examined for the parasitic infestation and observed the histological changes of internal organs.

### 2.2 Analysis of length–weight parameters

After collection, the samples were brought using polythene bags with water in live condition to the Disease Laboratory, Department of Fish Health Management, Khulna Agricultural University, Khulna‐ 9100, Bangladesh for investigation. the total length (l), and body weight (w) of the fish were recorded in data book.The length‐weight relationship were examined following Nasren et al. (2023), which is W=aL^b^. In where, 'W' represents the weight of the fish in gm, 'L' represents length of the fish in cm. The variables include 'a' for the intercept, 'b' for the weight at unit length (slope).

Condition factor (*k*) was calculated by employing the following formula:

$$k=\frac{100\times w}{l^{3}}$$

Where, *w* is the weight of the fish in grams; *l* is the length in centimetres.

### 2.3 Collection of parasites

The host fishes were anaesthetized by chloroform or by cutting at the neck region. A slit was made on ventral side near the genital pore on anal region and opened towards the head up to the opercular region. After careful opening, the stomach and the intestine were removed and put in a petri dish containing water for parasitic investigation. First, the outside part of stomach and intestine was washed then the stomach was opened using scissor for the investigation of parasite. and the parasites were kept in 10% saline for relaxation, flattened and fixed in fixative formaldehyde, alcohol, acetic acid (FAA).

### 2.4 Preparation of parasite for microscopic studies

The collected parasites were fixed in fixative FAA, stained in Borax carmine, cleared in lactophenol, and mounted in Canada balsam, according to Chandra (2008).

### 2.5 Histological slide preparations

The internal organ such as gill, kidney, liver, stomach and skin were taken carefully and perfectly preserved in 10% neutral buffer formalin for making histological slide preparations. The protocol of histological slide preparation during the experiment was followed as outlined by Rahman et al. (2023), Zaman et al. (2023), and the images were taken by using Optika light microscope, Italy (B9).

### 2.6 Identification and analysis of parasite

The Prevalence (*P*), Mean Intensity (MI), Abundance (*A*) and Index of Infestation (IOI) of collected parasites were identified followed bythe formula of Chandra (2008) and Ash et al. (2011).

(1) $$\mathrm{Prevalence}\;\left(P\right)=\frac{\mathrm{No}.\mathrm{of}\;\mathrm{host}\;\mathrm{fish}\;\mathrm{infested}}{\mathrm{No}.\mathrm{of}\;\mathrm{host}\;\mathrm{fish}\;\mathrm{examined}}\times 100$$

(2) $$\mathrm{Mean}\;\mathrm{intensity}\;\left(\mathrm{MI}\right)=\frac{\mathrm{No}.\;\mathrm{of}\;\mathrm{the}\;\mathrm{parasites}\;\mathrm{collected}}{\mathrm{No}.\;\mathrm{of}\;\mathrm{the}\;\mathrm{infested}\;\mathrm{hosts}}$$

(3) $$\mathrm{Abundance}\;\left(A\right)=\frac{\mathrm{No}.\;\mathrm{of}\;\mathrm{the}\;\mathrm{parasites}\;\mathrm{collected}}{\mathrm{No}.\;\mathrm{of}\;\mathrm{the}\;\mathrm{hosts}\;\mathrm{examined}}$$

(4) $$\begin{array}{ccc} & & \mathrm{Index}\;\mathrm{of}\;\mathrm{Infestation}\;\left(\mathrm{IOI}\right)\\ & & \;=\frac{\mathrm{No}.\;\mathrm{of}\;\mathrm{parasite}\;\mathrm{collected}\;\times \mathrm{No}.\;\mathrm{of}\;\mathrm{infected}\;\mathrm{hosts}\;}{\mathrm{No}.\;\mathrm{of}\;\mathrm{hosts}\;\mathrm{examined}} \end{array}$$

### 2.7 Statistical analysis

The collected data were recorded in microsoft Excel spreadsheet and the statistical analysis was done using IBM SPSS 17 (Chicago, USA) software.

## 3 RESULTS

**FIGURE 2:**
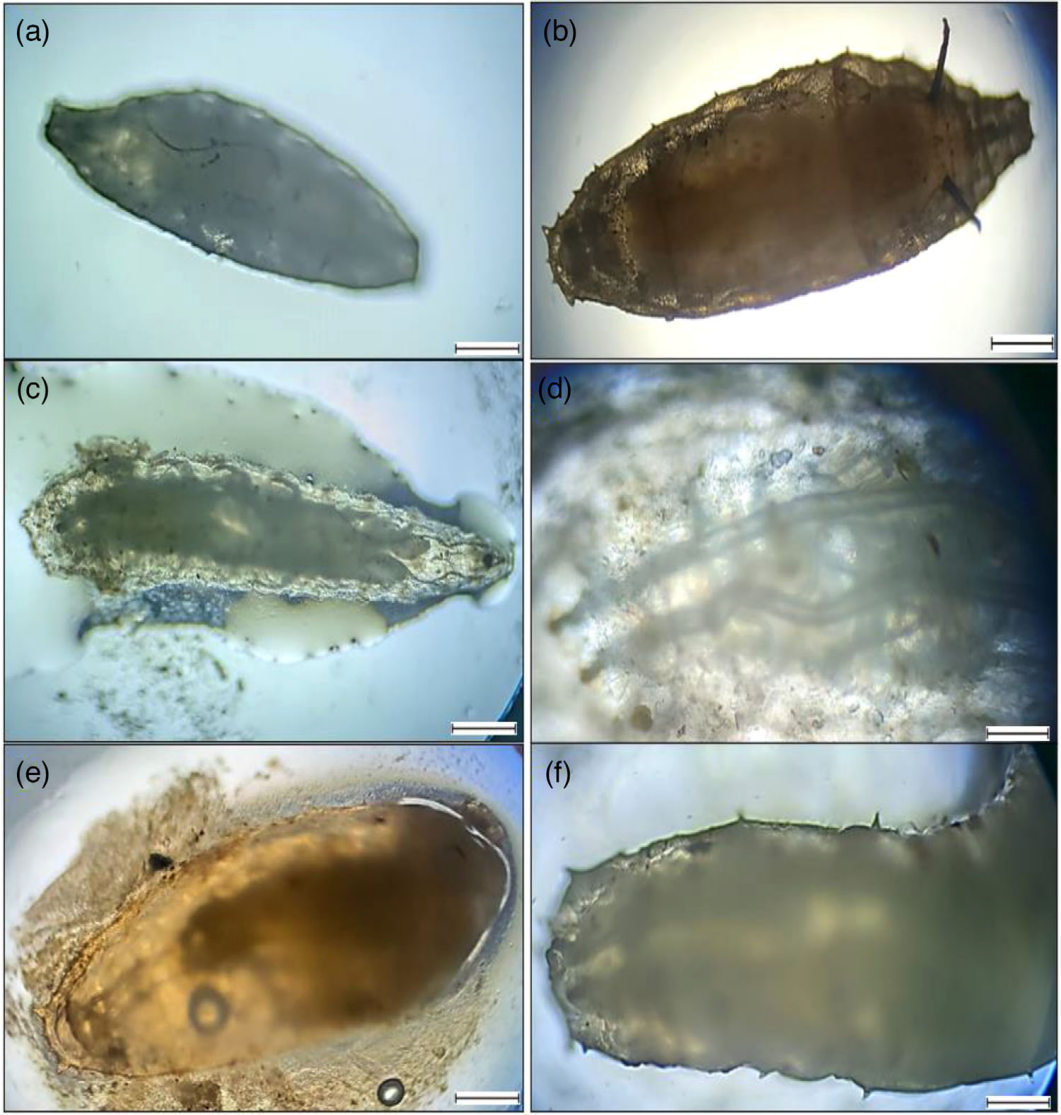
Identified parasites found in *L. rohita* using magnification 40×; scale 200 μm: (a) *Djombangia penetransbg*, (b) Cestode (unidentified), (c) Nematodes, (d) *Euclinostomum heterostomum*, (e) Digenia group (unidentified species), (f) Acanthocephala (unidentified).

**FIGURE 3:**
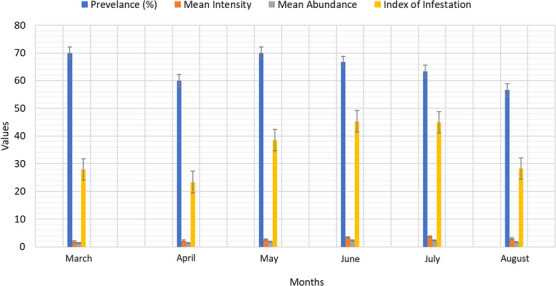
Comparison (Prevalence, Mean Intensity, Abundance and Intensity of Infestation) of parasitic infestation from the month of March 2023 to August 2023.

**FIGURE 4:**
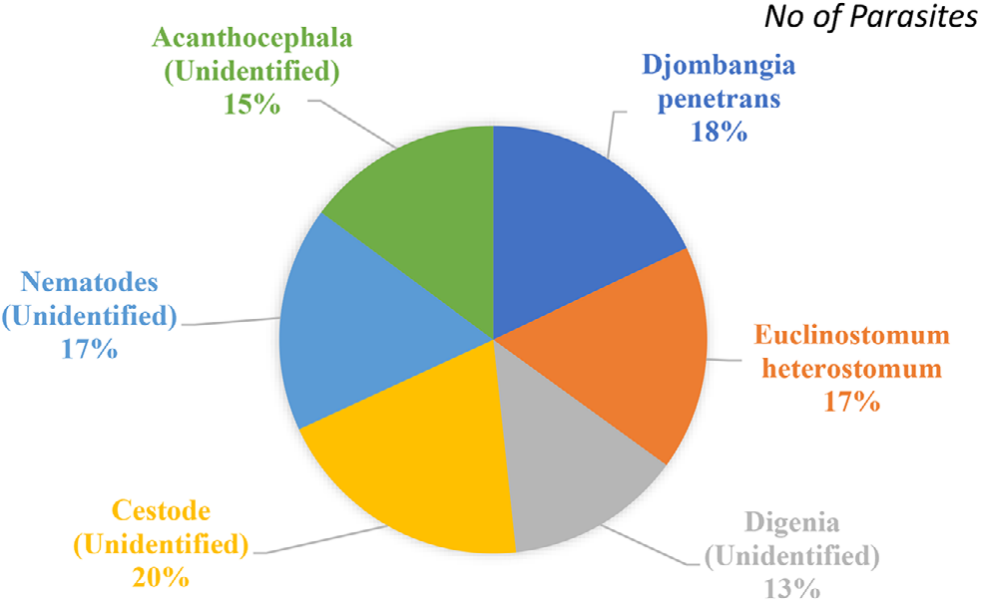
Different parasites with their count (%) in different groups.

**FIGURE 5:**
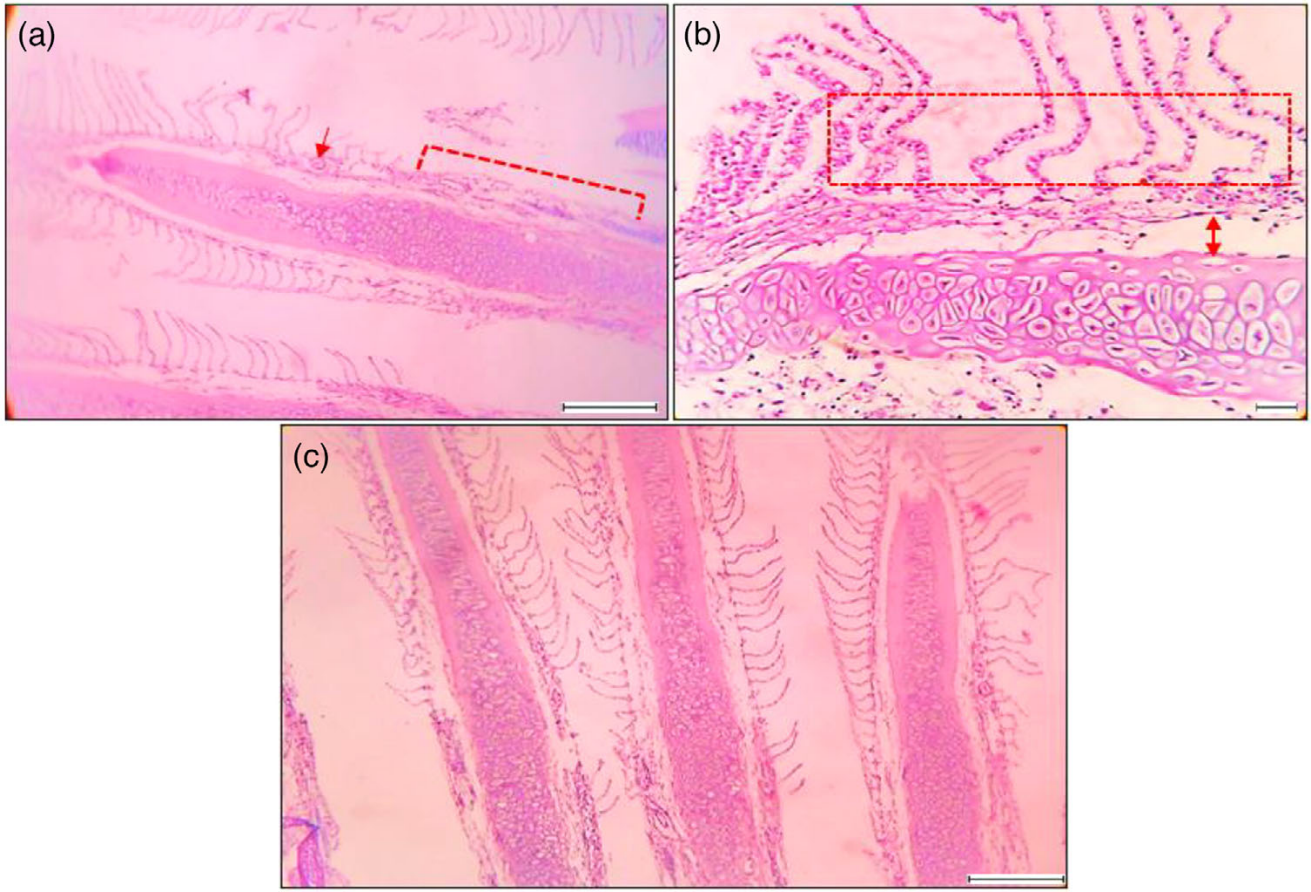
Histological features and alterations in gill using magnification 40×; scale 200 μm: (a) inflammation in secondary gill lamellae (arrow), missing secondary gill lamellae (area labelled); (b) secondary gill lamellae separated and remain detached from the gill arches (two side arrow), secondary gill lamellae become destabilized; (c) irregular and broken secondary gill lamellae.

**FIGURE 6:**
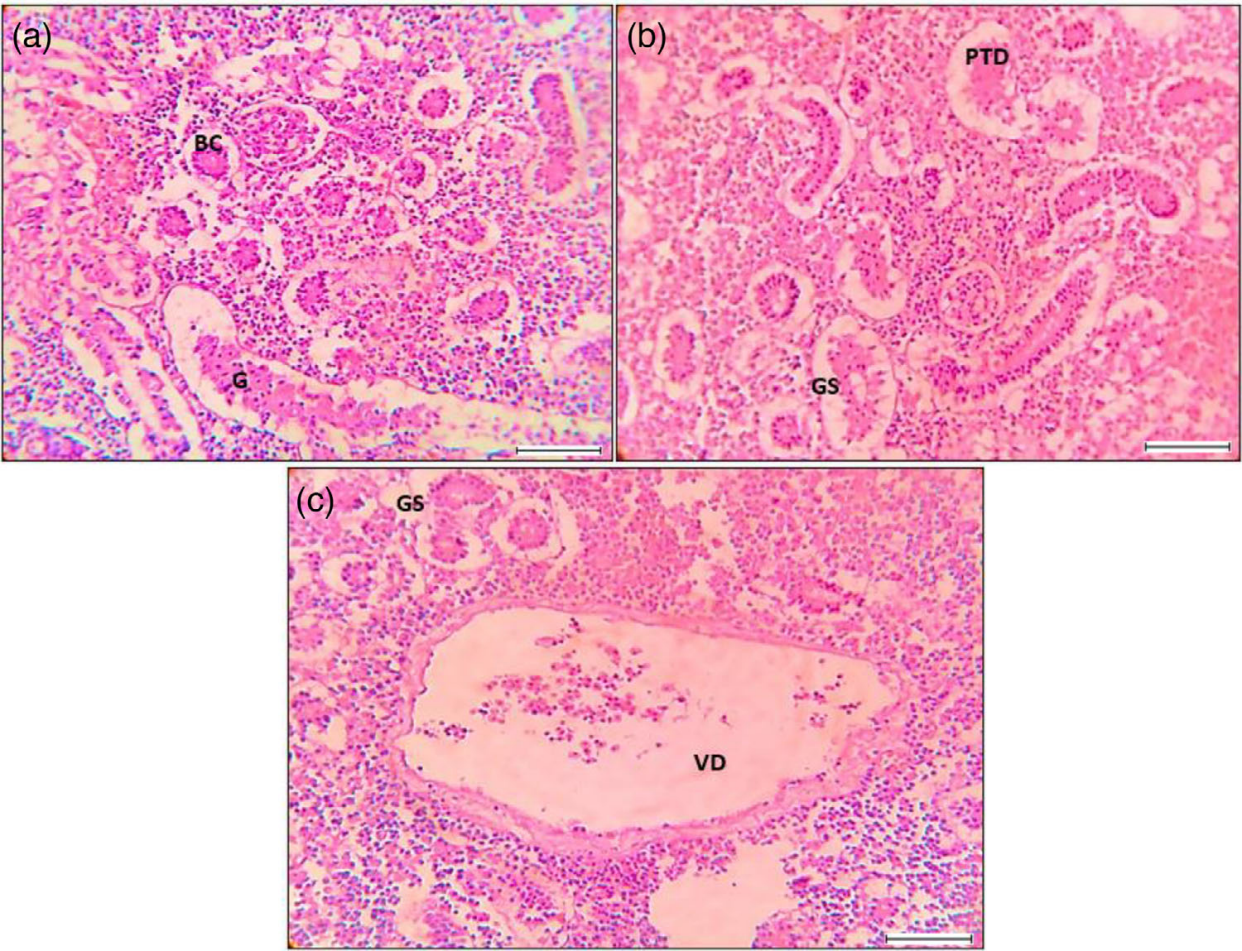
Histological features and alterations in kidney using magnification 40×; scale 200 μm: (a) Regular histological section of *Labeo rohita* containing Bowman's capsule (BC) and glomerulus (G); (b) malfunction observed in kidney histopathology with the presence of proximal tubule degeneration (PTD) and some extra presence of glomerular shrinkage (GS); (c) large vacuolar degeneration (VD) observed with glomerular shrinkage (GS).

**FIGURE 7:**
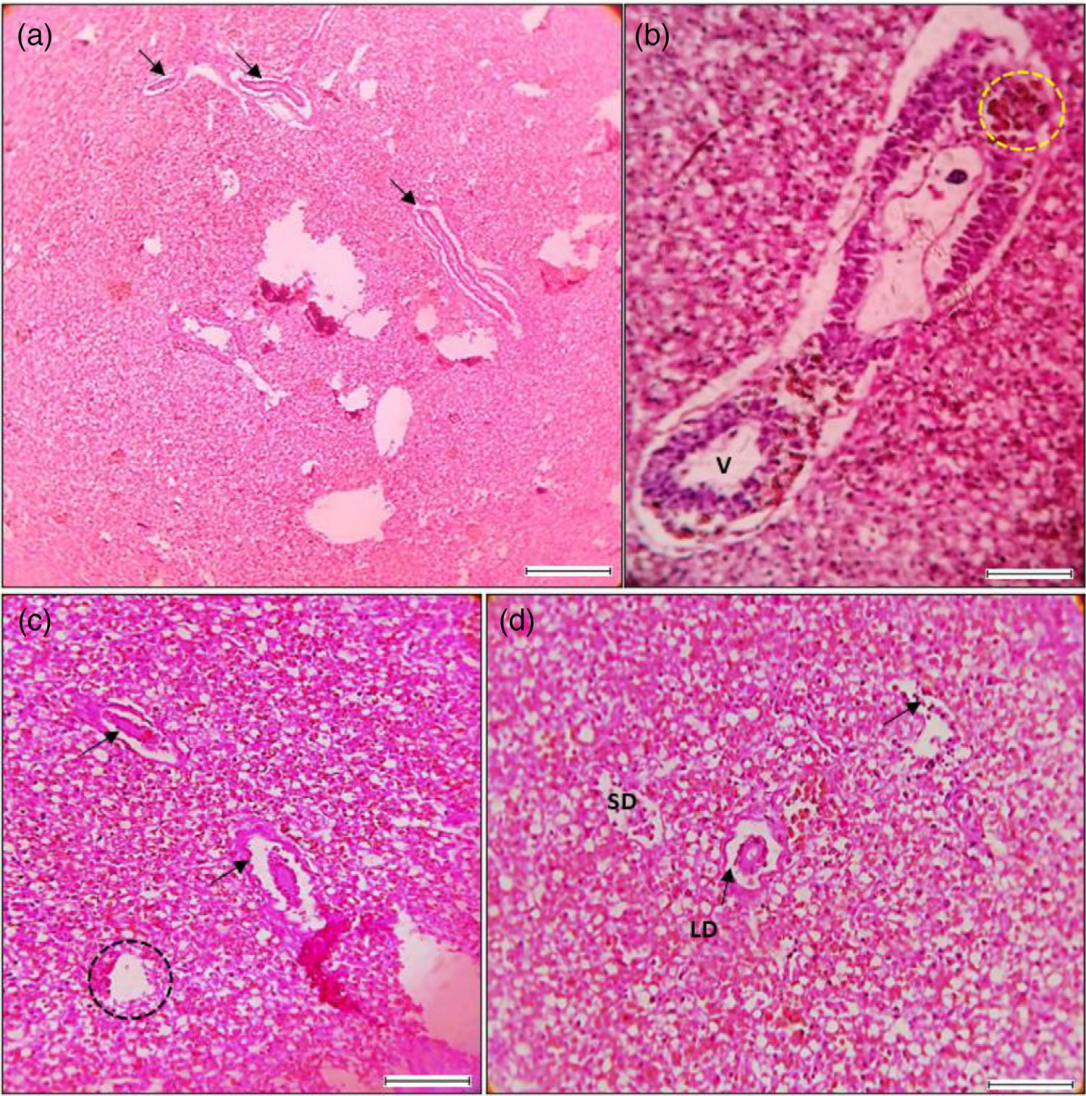
Histological features and alterations in liver using: (a) Presence of several parasite inside the liver sinusoid cells (arrow mark), (b) melano macrophage centre (MMCs) yellow circle zone and a peculiar zone of vacuole (V), (c) several zones of necrosis (arrow mark) and haemorrhages (black circle zone), (d) sinusoid dilation (SD) and presence of lipid droplets (LD).

**FIGURE 8:**
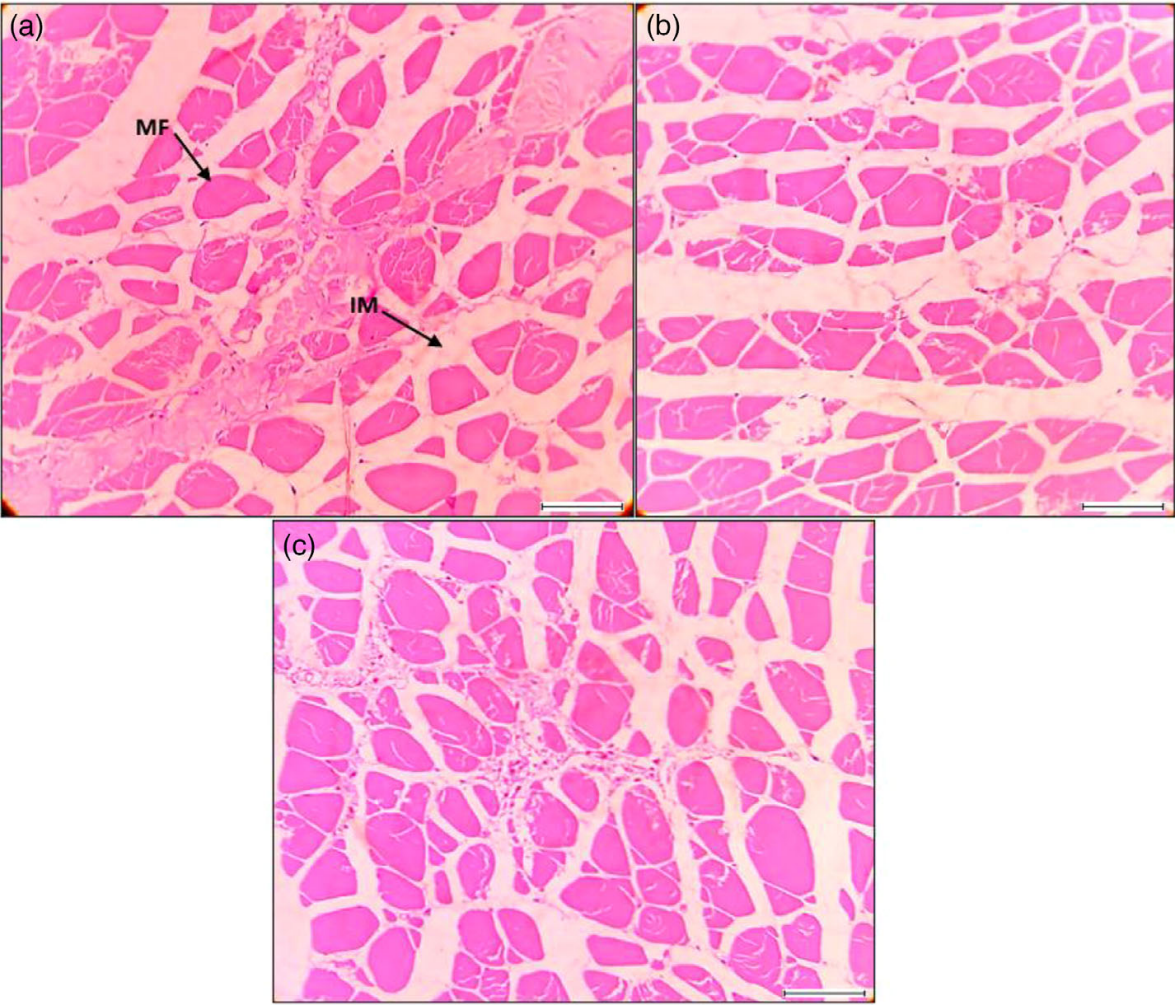
Histological features and alterations in skin using magnification 40×; scale 200 μm: (a–c) regular muscle histological features were observed during the experiment, myofibrils (MF); interstitial materials (IM).

**FIGURE 9:**
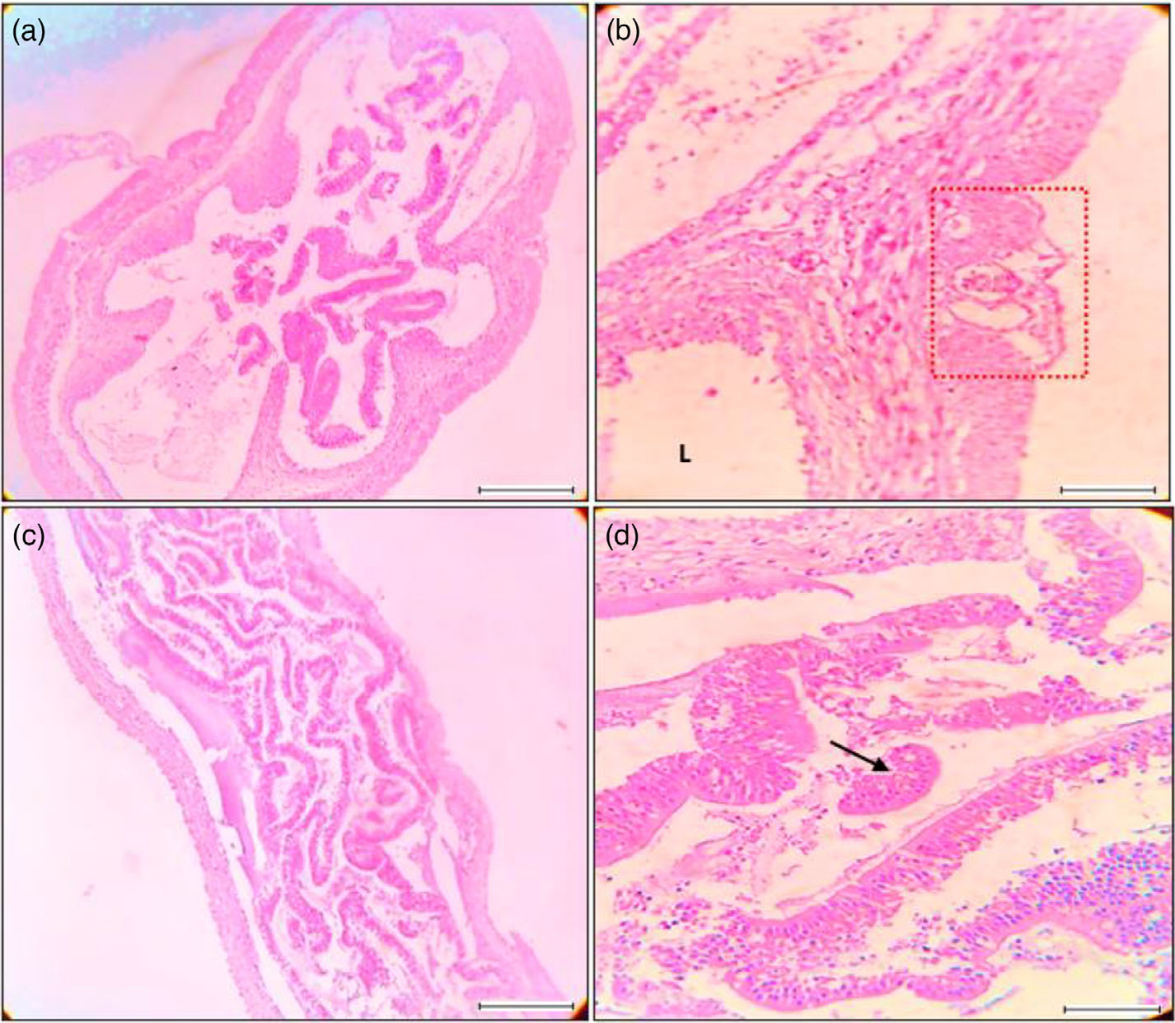
Histological features and alterations in stomach and intestine using magnification 40×; scale 200 μm: (a) section of intestine horizontal, (b) vacuolar degeneration in the mucosal layer (red zone), lumen (L) , (c) intestinal sectioning vertically, (d) disruption in Ville (arrow mark).

In Table 1, along with the variables, *a*, *b* and *k* make up a mathmatical growth model, the mean length and mean weight was 35.1 cm and 981.7 gm, respectively. The condition factor (*k*), relative condition factor (*k*_*n*_) and coefficient of determination (*R* ^*2*^) were 0.752, 1.0001 and 0.84, respectively, in where '*b*' not equal to 3 indicates allometric growth patters (Table1). A total of 180 *L. rohita* fishes were examined during the study period in where 116 fishes were spotted infested with a total number of 323 parasites of six groups (Tables 2). All the represented parasites were collected from *L. rohita*  fishes showing in Figure 2. Table 3 showed that the highest mean intesity (MI) was found in July with the value of 3.73 and the lowest was in March with the value of 1.90. On the other hand, the highest mean abundance (MA) was recorded in July (2.37) and the lowest was in April (1.30). According to index of infestation (IOI), the highest and lowest values was observed in June and April covering the value of 45.34 and 23.40, respectively (Table 3, Figure 3). The highest identified parasites was revealed in cestode group covering 20% and the lowest was in Digenia covering 13% of total infestation (Figure 4). Figure 5 presented that histological changes of gill lamella which was affected by identified parasites. Irregular and broken gill lamella was observed in affected gill of *L. rohita*. The presence of proximal tubule degeneration (PTD), some extra presence of glomerular shrinkage (GS) and large vacular degeneration (BD) were observed in kidney (Figure 6). In liver, sinusoid cell affected by serveral parasites, necrosis, hemorrhages and lipid droplets (LD) were observed (Figure 7). In skin, myofibrils and interstitial materials, irregular histological changes was developed (Figure 8) and disruption in ville & vacuolar degeneration in the muscosal layer was identified (Figure 9).

**TABLE 1:** Length‐weight relationship of *Labeo rohita* collected during the study period.

Sl. no.	Features	Values
01	Species	*L. rohita*
02	Size of sample	180
03	Mean length (cm)	35.1
04	Mean weight (g)	981.7
05	Parameter (*a*)	0.0011
06	Parameter (*b*)	3.66
07	95% CI of *a*	0.0008–0.0014
08	95% CI of *b*	3.568–3.759
09	Growth pattern	Positive allometric
10	Condition factor (*k*)	0.752
11	Relative condition factor (*k_n_*)	1.0001
12	Coefficient of determination (*R* ^2^)	0.84

**TABLE 2:** List of different parasites collected during experimental period.

Sl. no.	Group	Parasite name	Species confirmation	No. of parasites	Structure
1	Cestode	*Djombangia penetrans*	(Identified)	58	Oval
2	Trematode	*Euclinostomum heterostomum*	(Identified)	55	Tube
3	Digenia group	Digenia	(Unidentified)	43	Oval
4	Cestode	Cestode	(Unidentified)	64	Oval
5	Nematodes	Nematodes	(Unidentified)	55	Long rod shaped
6	Acanthocephala	Acanthocephala	(Unidentified)	48	Spiny round

**TABLE 3:** Parasitic infestation in *Labeo rohita* during study on the basis of months (180 days).

Months	Locations	SI	No of HE	ML (cm)	MW (g)	No of HI	PC	*P* (%)	MI	MA	IOI
March (2023)	Dumuria	Intestine	10	36.7 ± 1.34	1090.4 ± 2.26	6	13	70	1.90	1.34	28
	Paikgacha	Intestine	10	32.4 ± 2.21	1100.6 ± 1.34	7	12				
	Rupsha	Intestine	10	36.2 ± 1.66	1000.8 ± 2.36	8	15				
April (2023)	Dumuria	Intestine	10	38.7 ± 1.76	1020.7 ± 1.92	6	13	60	2.17	1.3	23.4
	Paikgacha	Intestine	10	33.2 ± 3.27	980± 4.63	5	17				
	Rupsha	Intestine	10	35.4 ± 2.68	920.6 ± 3.28	7	9				
May (2023)	Dumuria	Intestine	10	36.8 ± 1.66	1000.5 ± 2.38	8	15	70	2.61	1.83	38.5
	Paikgacha	Intestine	10	37.5 ± 2.82	1070.4 ± 1.86	7	18				
	Rupsha	Intestine	10	30.6 ± 1.37	1100.9 ± 3.44	6	22				
June (2023)	Dumuria	Intestine	10	33.4 ± 2.65	1200.2 ± 4.31	7	23	66.67	3.4	2.27	45.34
	Paikgacha	Intestine	10	38.2 ± 1.86	1230.4 ± 3.28	6	24				
	Rupsha	Intestine	10	38.3 ± 2.21	1100.7 ± 4.63	7	21				
July (2023)	Dumuria	Intestine	10	31.3 ± 1.63	1050.4 ± 3.64	8	19	63.34	3.73	2.37	44.97
	Paikgacha	Intestine	10	39.1 ± 1.43	1040.8 ± 3.36	6	30				
	Rupsha	Intestine	10	33.7 ± 2.21	1000.7 ± 2.76	5	22				
August (2023)	Dumuria	Intestine	10	32.6 ± 1.63	890.8 ± 4.36	4	13	56.67	2.94	1.67	28.34
	Paikgacha	Intestine	10	31.2 ± 1.78	960.4 ± 4.78	7	25				
	Rupsha	Intestine	10	37.5 ± 2.58	1050.7 ± 3.64	6	12				

Abbreviations: HE, Host Examined; HI, Host Infected; IOI, Index of Infestation; MA, Mean Abundance; MI, Mean Intensity; P, Prevalence; PC, Parasite Collected; SI, Site Infected.

## 4 DISCUSSION

The length weight relationship of*L. rohita* showed positive allometric growth which indicate the different growing rate of body part. Rahman and Parween (2001), Alam et al. (2006) and Rahman et al. (1998) reported comparable parasitic infestations in various freshwater fish species within Bangladesh. A condition factor of 1 indicates that an organism possesses adequate nutrition and is in an environment conducive to its well‐being (Ujjania et al., 2012). Naeem and Salam (2005) similarly found a b value of 3.32 for bighead carp (*Aristichthys nobilis*), indicating satisfactory growth conditions. In the study, 180 *L. rohita* were examined, revealing the presence of six distinct parasite group, consisting of endoparasites. Most parasites were collected from the gastrointestinal system of fish, with a particular focus on the intestine and stomach regions. Numerous studies, including those by Harvey and Thompson (1995); Otto and Mackauer (1998); as well as Paine et al. (2004), provide compelling evidence suggesting that parasites exhibit a preference for hosts based on their superior body size and nutritional quality. Arthur and Ahmed (2002) compiled a comprehensive list of parasites infecting fish in Bangladesh, observing that *L. rohita* commonly hosts a variety of pathogens, including protozoans (*Ichthyophthirius multifiliis* and *Trichodina* sp.), Myxozoa (*Myxobolus* sp. and *Thelohanellus dogieli*), Digenea (*Neascus* sp. metacercaria), monogeneans (*Dactylogyrus* sp.), Acanthocephalans (*Echinorhynchus* sp.) and Branchiura (*Argulus* sp.). Additionally, Khan et al. (2003) identified *Lernaea cyprinacea* Linnaeus, 1758, and *Rhabdochona charsddensis* (nematode) infestations in the gills and gut of *L. rohita*, respectively, at Meinhart and Mini dams in the Potohar region of Pakistan. More recently, Bhuiyan et al. (2007) documented the presence of protozoans (*Trichodina*, *Chilodonella* and *Myxobolus*), monogeneans (*Dactylogyrus* and *Gyrodactylus*), Digenea (*Eucreadium*), nematodes (*Camallanus*) and Crustacea (*Argulus*) parasites in *L. rohita* specimens collected from various water bodies in the Rajshahi district of Bangladesh. Shomorendra et al. (2005) documented seasonal variations in the prevalence of helminth parasites across four fish species, including *Channa gachua*, *C. striatus*, *Clarias batrachus* and *Anabas testudineus*. Their findings revealed fluctuations in the occurrence of five parasite species: one digenetic trematode (*Astiotrema reniferum*), one nematode (*C. anabantis*), one acanthocephalan (*Pallisentis ophiocephali*) and two cestodes (*Djombangia penetrans* and *Capingentoides singhi*). Farhaduzzaman et al. (2009) conducted a study on parasite prevalence in the Indian major carp *L. rohita* within the Rajshahi district of Bangladesh. Their research revealed the presence of ten parasite species, with seven identified as ectoparasites and three as endoparasites. Among these, the helminth parasites included five species: *Dactylogyrus vastator* Nybelim, 1924, *Gyrodactylus elegans* Nordmann, 1832, *Camallanus ophiocephali* Nicoll, 1909, *Fellodistomum agnotum* Pearse, 1933 and *Eucreadium* sp.

The findings of this study suggest that the highest incidence of infection occurred during the rainy season, whereas the lowest occurred in winter. This pattern can be attributed to increased food availability and higher temperatures, which prompted greater fish movement and created a more polluted environment conducive to infestation/infection. Additionally, the level of parasitization displayed a notable positive correlation with the size of the fish, with smaller fish showing fewer parasites, likely reflecting their feeding habits (Upadhyay et al., 2012). The findings regarding parasitic infestations align with previous studies conducted by Farhaduzzaman et al. (2009) and Mofasshalin et al. (2012) in carp fish populations of Bangladesh.

In this study, gill, kidney, liver, skin and gastrointestinal tract affected by parasitic infestation. Barassa et al. (2003), Eiras et al. (2008, 2009) and Adriano et al. (2009) conducted histological examinations of gills afflicted with myxozoan parasites. Their analyses revealed the presence of numerous large cysts within the gill filaments, yet no significant inflammatory reaction was observed at the site of infection which is similar to our findings. The intensity of parasites within various organs of *L. rohita* exhibited seasonal variations, with higher levels recorded during winter. This period has been consistently identified in previous studies (Akter et al., 2008; Farhaduzzaman et al., 2009; Mofasshalin et al., 2012) as a time of increased susceptibility of fish to parasitic infestations. Several authors, including Ashry et al. (2013), Tayel et al. (2014), Kadry et al. (2015), Mahmoud and Abd‐El Rahman (2017), Bayomy and Mahmoud (2007), Tayel et al. (2018) and Ahmed et al. (2019), have also observed similar alterations in the liver, kidney and gills. They attributed these malformations to industrial, agricultural and sewage wastes, which can alter water quality and lead to parasitic infections. Fish skin and gills serve as primary sites for parasitic infections, acting as the first line of defence against various parasitic diseases by secreting mucus, thereby restricting pathogen proliferation (Nachev et al., 2013). Several studies have highlighted the capacity of various parasites to accumulate heavy metals within their bodies. Consequently, nematodes, cestodes and even Acanthocephalan species have been proposed as potential indicators of water and tissue heavy metal toxicity (Baruš et al., 2007; Palíková & Baruš, 2003). However, despite these findings, our understanding of the relationship between parasites and toxicity remains incomplete (Nachev et al., 2013). Therefore, ensuring a consistent supply of high‐quality water for pond construction is imperative to uphold optimal growth rates and product quality (Agoz et al., 2005). The histological results obtained in this study corroborate previous findings suggesting that parasites can inflict significant damage on the internal organs, such as the liver and musculature, of various fish species (Adeyemo & Agbede, 2008; Shareef & Abidi, 2012).

## 5 CONCLUSION

The investigation of parasitic infestation in *L. rohita* in south‐western Bangladesh revealed a significant threat to fish health and ecosystem sustainability. The prevalence of parasites not only affects individual fish but also disrupts the overall aquatic ecosystem and food chain balance. This underscores the urgent need for collaborative efforts among researchers, policymakers and local communities to address this issue. By understanding and mitigating parasitic infestation, we can work towards preserving the ecological balance and ensuring the long‐term viability of fish populations in Khulna district of Bangladesh.

## AUTHOR CONTRIBUTIONS

*Conceptualization; methodology; project administration; data curation; supervision; writing – original draft; writing – review and editing; funding acquisition; investigation*: Basir Ahammad. *Methodology; validation; formal analysis; writing – original draft; writing – review and editing*: Bhaskar Chandra Majumdar. *Writing – original draft; writing – review and editing; validation; formal analysis; visualization*: Angkur Chowdhury. *Data curation; Writing – original draft; Writing – review and editing; Software*: Rasel Mia. *Visualization; writing – review and editing; writing – original draft; resources*: MD Zobayer Rahman.

## CONFLICT OF INTEREST STATEMENT

Authors declare no conflicts of interest in this research work.

### ETHICS STATEMENT

The study was performed using ethical recommendations of the government of People's Republic of Bangladesh.

### PEER REVIEW

The peer review history for this article is available at https://www.webofscience.com/api/gateway/wos/peer-review/10.1002/vms3.1489.

## Data Availability

The data that support the findings of this study are available from the corresponding author upon reasonable request.
